# Supplementary material for: Are carbon emissions trading and green financial instruments synergistic? -Comprehensive quantitative research based on content analysis
Source: PLoS One. 2024 Mar 7;19(3):e0298601. doi: 10.1371/journal.pone.0298601 (PMC10919601; doi:10.1371/journal.pone.0298601)
Supplement: S1 Data — (ZIP) [file pone.0298601.s001.zip › Data/地级市Z_F工作报告.zip!/╣π╢1⁄2/╟σ╘╢╩╨/└┤╫╘╣1⁄2╓┌║┼í╛┬φ┐╦╩2╛▌═°í┐_2013.docx]

各位代表：

现在，我代表市人民政府向大会报告政府工作，请予审议，并请政协各位委员和其他列席人员提出意见。

2012年工作回顾

刚刚过去的2012年，是按照“桥头堡”战略谋篇布局、凝心聚力的一年。在市委的正确领导下，在市人大和市政协的监督支持下，我们紧紧围绕市委的决策部署，以富民强市为目标，突出交通、产业、城市、区域、民生五大重点，围绕重大基础设施建设、主体功能区建设、产业发展平台建设、城市扩容提质和基本公共服务均等化等关键工作，加大改革、投入和推进力度，实现了市政府工作的良好开局。

刚刚过去的2012年，是沉着应对严峻复杂形势的一年。在国际国内发展环境复杂多变，房地产和基本建设持续萎缩，企业经营成本不断上升的情况下，我市发展方式粗放、产业结构不合理、经济抗风险能力不强等问题更加凸显，经济发展遭遇新世纪以来最大困难和挑战。面对严峻的发展困境，我们把握稳中求进的总基调，采取了一系列稳增长的举措，促进了经济平稳健康发展。初步统计，全市实现国内生产总值1029亿元，同比增长5.1%。人均国内生产总值2.74万元，增长4.3%。来源于清远的财政总收入207.5亿元，其中全市公共财政预算收入86.9亿元，增长3%。

刚刚过去的2012年，是转型发展取得良好开端的一年。我们坚持从容发展、科学发展的理念，着力转变经济发展方式，脚踏实地调整产业结构，经济发展质量有效提升。工业增加值率由16.7%提高到20.9%，整体质量得到了提升。财政收入结构得到优化。城镇居民人均可支配收入19514元，增长10.5%；农村居民人均纯收入8612元，增长16%；扣除价格因素实际增长分别达到7.4%和12.7%，明显快于全市经济增长速度，更好地体现了改善民生的发展宗旨。

过去的一年，我们主要抓了以下八项工作：

一、积极应对经济发展困境

加大政府主导的投入。启动了95项总投资达1847.4亿元的重点项目建设，带动了社会和企业投资。积极推进政银合作,通过BT、BOT等融资模式，成功吸引一批大型企业参与我市基础设施建设，确保了重点项目建设投资。全市重点项目完成投资198.9亿元，完成率比上年提高了9个百分点。

积极为企业排忧解困。建立市县领导走访企业制度和政企沟通联系机制，市县两级共召开70多次政企沟通会和协调会，帮助企业解决实际问题300多个。积极扶持企业上市，5家企业进入上市“绿色通道”，8家企业成为上市后备。改革企业登记制度，认真落实减税政策，大力清理和减免涉企收费，切实减轻企业负担。设立政府信用担保基金，积极为中小微企业提供融资担保服务，全市贷款余额比年初增加106.5亿元，存贷比由年初的54.6%提高到59%。

大力促消费稳出口。在珠三角主要客源地举办旅游推介会，全年接待游客2820.9万人，旅游总收入178.7亿元，分别增长8.4%和10.7%。积极为企业开拓市场搭建平台，全市社会消费品零售总额459.6亿元，增长10.7%。着力构建便利高效的通关环境，全年完成外贸出口24.4亿美元，增长4.2%。

集中财力保民生。着力压减一般性财政支出，加大民生工程投入力度，全年财政民生投入117亿元，增长12.23%，增速比公共预算支出高3.23个百分点，民生投入占公共预算支出比重由上年的66.2%提高到68.2%。

二、全力抓好关系全局的基础工作

突破了重大基础设施建设瓶颈。一度制约基础设施建设的征地拆迁以及与花都区的工作协调等瓶颈问题，在去年的工作中取得了突破，基础设施建设进入快车道。广清城际轨道项目正式动工，我市将成为粤东西北地区首先拥有城际轨道的城市。广乐、二广及广清高速扩建清远段的控制性工程基本完成，路基工程完成60%；佛清从高速北段征地红线图和施工图基本完成；连佛高速正抓紧工程立项审批。清三一级公路大修实现单边通车，清佛公路大修首期工程完工，银英公路扩建、石龙大道、清连二级公路大修工程基本完成。106国道佛冈段、354省道清新段等10条国省道和县道大修工程开工建设。北江五桥前期工作基本完成。省天然气管网工程清远段完成68公里管道和清远末站建设。清新抽水蓄能电站上库工程基本完工，下库、水道、厂房等工程完成55%以上。华润连州风电一期工程建成并网发电。输变电工程建成投产110千伏及以上输电线路58.1千米，增加变电容量10万千伏安。一批新的电网建设和变电站工程及北部农网改造，纳入南方电网投资计划。清远水利枢纽主体工程基本完工，大燕河整治工程完成年度投资计划。第二、第三新水厂的建设全面启动。3G通讯、无线宽带快速发展。

理顺了市区土地等历史遗留问题。完善了政府与企业合作开发土地的方式，共处置30宗合作开发合同，涉及土地面积25.3平方公里（折合3.8万亩），既坚守了政府信用，又避免财政风险。全面清理闲置用地，加大“三旧”改造力度，共为119宗、7402亩历史用地完善了手续。将园区开发模式由“引凤筑巢”调整为政府主导、利用社会资金进行配套建设，通过整合、回购、新征用等方式，新规划统筹了20平方公里（折合3万亩）市级工业园区用地。

完善了指导长远发展的各类规划。完成了中心城区总体规划修编和分区规划、专项规划、重点片区控制性规划。重新调整和修编了中心城区土地利用总体规划。完成了8个县（市、区）主体功能区规划。修订了产业发展规划，出台了旅游、公交、信息化等13项专项发展规划。开展了乡村规划工作，6个示范村的规划已完成。

建立了人才引进及主体功能区配套政策。制定了人才发展规划、人才队伍建设、区域人才协调发展等一系列吸引和培养人才的政策措施，与广州市、广东老教授协会、中南大学、暨南大学等签订了人才培养引进合作协议，与东莞市搭建了招才引智合作平台，出台了市政府顾问聘用管理办法。根据差异化发展的定位，建立了财政资金差异化配套和生态补偿机制，调整完善了市对县的科学发展观绩效考核评价体系。

三、扎实推动产业优化升级

改造提升传统优势产业。制订了建筑陶瓷和再生金属产业转型升级方案，出台了再生金属产业技术路线图，启动了陶瓷产业“清洁生产”计划。继续抓好中心区域废旧物资拆解场清理整治，引导金属拆解业集聚发展，清城区有色金属再生产业集群升级示范区在省考核中被评为优秀等级。企业技术创新取得新进步，豪美铝业技术中心获准成为我市首家国家级企业技术中心。

加快培育新的支柱产业。启动了莲湖产业园、光电产业园和“华南声谷”的规划建设，佛山（清远）产业转移园扩园方案获省批准，佛冈县部分园区纳入省级产业转移园，民族工业园升格为省级产业转移园，一批优质项目正式落户。交通银行、汇丰银行等12家金融、保险、证券机构在我市新设立了分支机构，结束了清远过去十多年未有新的金融机构进驻的历史。出台了鼓励总部企业发展的若干规定。与广州、东莞、佛山、郴州等城市签订了合作协议。几个投资规模大、技术含量高的重大产业项目，已经确定了落户清远意向，目前正在做最后的落实工作。全市共引进新项目254个，合同投资511亿元，累计实际投入105.2亿元。其中亿元以上项目114个，合同投资456.57亿元。

淘汰落后产能取得突破。强化环保、用地、财税等指标约束，按项目优劣安排用地指标，预计单位建设用地产出GDP同比增长4.0%。清理涉重金属作坊230家，关闭小作坊126家，关停落后企业33家，拒绝43个高耗能、高污染项目落户，完成了省下达的节能减排目标。清理268宗违法开采矿山案件，关停108家矿场，顺利通过2011年卫片执法检查。国家重点生态工程建设基本完成，连州、佛冈成功创建为省林业生态县。

四、大力改革困扰发展的体制机制

调整优化管理体制。将城市综合管理职能及园林、环卫等管理事务全面下放给清城区，将部分市级经济管理权下放高新区，对华侨工业园和民族工业园实行属地管理，向顺德清远（英德）经济合作区转移市级经济管理事项51项。调整优化中心区域财政分配体制，理顺市中心区办学体制。完成了中心区域学校和幼儿园布局调整，预计2013年市区将增加幼儿园至高中学位2.4万个。

创新投融资体制。完善了政府土地储备制度，实行土地开发储备“一个口子”进出，增加了近万亩政府储备土地，保障了城市建设的良性循环。成立了城市建设、水务、旅游发展等三家投融资公司，实现了由政府直接投融资向企业化、市场化运作转变。

改革行政运行体制。对35个市直单位1294项审批事项进行了清理，公布市级政府第一批行政审批事项改革目录，取消、下放和向社会转移审批事项249项，审批事项压减40.4%。深化人事和收入分配制度改革，行政、事业单位用人招录工作更加公平公正。建立中层干部公开竞争选拔制度，市直单位全员竞岗顺利完成。实施非税执收单位经费供给改革和基金管理改革，在全省各地市率先构建集“预算编制、预算执行、监督检查、绩效评价”于一体的财政大监督工作机制。全年新登记社会组织88个，市旅游协会等一批社会组织完成“去行政化、去垄断化”改革。

五、加快区域中心城镇建设

提升中心城区建设水平。提出了“绿色湖城”的建设构想，确立了人本城市的理念，并在城市功能定位、规划、交通及产业发展上主动对接大广州。调整优化了“四馆合一”、北江五桥、全民健身广场和一批城市道路的规划设计。作为省确定的粤东西北地级市城区扩容提质试点，启动了燕湖新城、江北新区、银盏新市镇建设，铺开了省级职教基地、莲湖工业园的配套建设。首批38个总投资达92.1亿元的市政项目正在加快推进，第二批44个总投资约200亿的项目即将动工。清新撤县设区挂牌运作。

推进次中心城市建设。英德市基本完成了“四镇合一”城市总体规划的修编，启动了大站东区和长岭片区建设，高铁客运站投入运营，档案馆、科技馆等一批项目建成，月桂湖、北江三桥、迎宾大道等项目加紧推进。连州市已经完成城市总体规划修编，城西核心区控制性详细规划正在编制，新区建设全面铺开，兴连大道等一批市政工程加快推进。

着力完善城镇功能。新（改）建公园11个，绿道154.1公里，建成区绿地率达34.9%。动工新建污水处理厂3家，完成13.5公里截污主干管网建设。市区青山垃圾场扩容工程项目顺利完成，各县（市、区）新建生活垃圾中转站9个。城镇生活污水处理率、生活垃圾无害化处理率分别达到75.6%和51.5%。积极推进国家园林城市创建工作，加快县城和中心镇市政道路和公共服务设施建设，城市美化、绿化、亮化水平进一步提升。

六、加大农业发展和农村建设力度

“两品”工程成效明显。全市新增有机农产品30个、绿色食品8个、广东省名牌产品（农业类）2个、省级食用农产品质量安全示范点76个、无公害农产品产地认定企业9家、产品10个。省级重点农业龙头企业发展到16家，新登记农民专业合作社261个，出栏量500头以上的规模生猪养殖场达1119个。14宗农田水利小型灌区改造工程全面完成，清新、英德成为全省农田水利建设示范县，各县（市、区）均被列入全省小型农田水利建设重点县。全市农林牧渔业产值234.9亿元，增长5.3%。

农村面貌持续改善。4个镇创建成为省宜居示范城镇，7个村庄创建成为省宜居示范村庄，佛冈龙南片区新农村实验区示范点住房改建初显成效。共完成新农村公路路面硬化工程540公里，农村电网改造工程全面铺开，村村通自来水工程正式启动。21个乡镇农民体育健身场所建成投入使用。实施农村清洁工程，推进垃圾定点堆放、定期清理。以改革基层政务服务方式为突破口，推进农村综合改革，53个镇级服务中心、460多个村级服务中心投入使用，分别占镇（街）、村委总数的68.8%和48.4%。阳山县农村综合改革走在全省前列，被省作为典型向粤东西北地区推广。

扶贫“双到”任务全面完成。302个省定贫困村全年共落实帮扶资金9.02亿元，培训贫困劳动力4.3万人。建立幸福安居示范村36个，累计建立互助金试点村252个、脱贫示范村100个。省定贫困村集体经济收入平均达到15万元，贫困人口年人均纯收入达到9200元。完成“两不具备”移民搬迁安置4514户20775人、农村低收入困难户住房改造9244户，超额完成省下达的任务。

七、认真办好社会关注的民生实事

公共民生事业加快发展。出台并启动推进教育、医疗卫生基本公共服务均等化实施方案。成功创建省教育强镇17个，申报省教育强县（市）2个。学前教育三年行动计划顺利推进，完成41所义务教育规范化学校、29所乡镇中心幼儿园、30所村级幼儿园的建设工作，启动了中心城区一批中、小学校建设，义务教育规范化学校覆盖率达82.3%。“普高”成果进一步巩固，高中阶段教育水平稳步提高，高等教育、特殊教育稳步发展，民办教育和成人教育规范发展。医疗卫生体制改革进一步深化，市直医院服务能力有效提升，基层医疗机构标准化建设成效明显，卫生监督和疾病预防控制工作扎实推进。全市“三馆一站”实现免费开放，基本完成20户以下已通电自然村广播电视“村村通”工程，农村文化基层设施全覆盖工程有序推进，广东瑶族博物馆正式挂牌。完成了全市非物质文化遗产资源普查工作，制订了保护和开发规划，连州慧光塔、连南南岗古排保护项目被列入国家文化和自然遗产保护设施中央预算内投资计划。CBA赛事进入清远，恒大皇马足球学校顺利开学。计划生育工作得到加强，户籍人口自然增长率控制在8.5‰以下。

基本民生保障得到加强。超额完成养老、失业、医疗、工伤、生育保险扩面任务，3个县实现了城乡居民社会养老保险全覆盖，全民医保目标基本实现，社保基金累计结余68.5亿元。全年救助低保对象13.2万人，低保金支出1.7亿元，实现了应保尽保。解决了2575户家庭的住房困难问题，完善了保障性住房准入和退出机制。

热点民生问题有效解决。落实种粮、水稻良种和农机购置补贴2.67亿元，申报“一事一议”财政奖补项目1329个，总投资6.2亿元，受益人口超过89万。全市新增城镇就业5.67万人，高校毕业生初次登记就业率98.47%，农村劳动力转移就业7.24万人，经过技能培训后转移就业2.26万人，城镇登记失业率下降到2.75%，零就业家庭保持动态为零。及时向低收入群众发放临时价格补贴，居民消费价格指数上涨控制在3%以内。实行60岁以上老人免费乘坐市区公交政策。加强了食品药品市场的安全监管。

十件民生实事基本完成。除群众艺术馆及北江市区段绿道因规划设计调整延后建设外，其余八件民生实事已全部完成。率先在粤北地区为80岁以上高龄老人发放津贴；飞来湖周边绿道基本建成；完成了10个样板肉菜市场的升级改造，新启动了一批市场改造项目；中心城区新增公交线路5条，延伸和调整公交线路8条，新投放公交车38台、出租车100辆；完成中心城区主干道交通安全设施建设，市区交通秩序明显好转。

“三打两建”和应急维稳工作成效明显。共查处欺行霸市刑事案件1715宗，打掉团伙268个；查处制假售假案件5280宗，捣毁制假售假窝点890个；查结商业贿赂案件368件，处理涉嫌“保护伞”173人。“两建”试点扎实开展，政府机关部门全部安装使用正版软件。认真抓好综治维稳和信访工作，社会大局保持稳定，加强社会治安管理成效明显，有效警情和“两抢一盗”案件发案率分别下降13.26%和9.62%。积极开展安全隐患排查，妥善处置了英德8·27爆炸事故等突发事件。大力推进应急体系建设，应急管理水平有效提升。

八、切实加强政府自身的改革和建设

加强民主法治建设。自觉接受人大、政协的监督，共办理人大代表议案、建议和政协委员提案286件。完善人大代表约见市长、市政府与市政协无主题会议制度，广泛听取社会各界的意见和建议，鼓励支持人民群众、新闻媒体对政府工作进行监督。建立了政府新闻发言人制度，对首批新闻发言人进行了业务培训。修订了政府工作规则，完善了政府法律顾问、重大决策专家咨询等制度。“六五”普法活动深入开展。成立了市县两级预防腐败局，建立了廉政风险防控长效机制，查处损害群众利益的案件325起。

推进机关作风建设。积极开展以治理“庸懒散软”为重点的“正风”行动，组织开展明查暗访和“公述民评”活动，共对275个单位进行明查暗访488次，制作机关作风暗访专题片12部，共377人（次）被问责。畅通投诉举报渠道，共受理行政效能投诉118件，办结率达100%。大力推行服务承诺制、首问负责制和限时办结制，机关服务质量得到提升。

强化行政效能建设。启动“工作落实年”行动，建立重大项目跟踪服务制，开通重点项目办事“绿色通道”，大力推进现场办公，精简和优化前置审批，将重复、相近、多环节的审批事项整合归并，取消可通过事后监管达到管理目的审批事项。加快网上办事大厅建设，43个部门389项业务进驻网上办事大厅，177项业务开通了在线申请功能，“一网式”审批率达55.7%，比上年高出近一倍。

我们还扎实推进各项社会事业发展，妇女、儿童、老龄、人防、档案、气象、方志、外事、侨务、对台等工作取得新成绩；国防双拥和民兵预备役工作进一步加强；民族、宗教关系保持和谐。

各位代表，过去一年各项成绩的取得，是全市上下在市委正确领导下，同心同德、克难求进、团结拼搏的结果，也是方方面面关心支持的结果。在此，我代表市人民政府，向辛勤工作在各个领域的全市人民，向市人大代表、政协委员，向市各民主党派、工商联、人民团体、社会各界人士和离退休老同志，向驻清人民解放军、武警官兵、公安政法干警和部省驻清单位，向所有关心、支持、参与清远建设和发展的中外投资者、科技工作者、异地务工人员和海内外朋友们，致以衷心的感谢和崇高的敬意!

在梳理工作成效的同时，我们也认真分析了政府工作中存在的突出问题：一是，经济工作还存在很多薄弱环节，帮扶企业的力度还不够大，对企业普遍存在的招工难和引进优质人才难的问题，还缺乏针对性的解决办法，对重点项目没有建立一套抓落实的有效机制，对县（市、区）的经济发展缺乏分类指导和具体支持。二是，科学民主的决策机制在实际工作中还落实不到位，一些项目策划不够科学，论证不够充分，社会的参与度不高。三是，政府职能部门和工作人员服务意识不强、办事效率不高、工作作风不实等问题仍然不同程度地存在。四是，对群众反映强烈的民生问题，还缺乏认真的梳理，还没有拿出系统的解决办法。五是，安全生产监管责任还落实不到位，基层政府环境保护意识还不强，安全生产和环境事件较上年多发。对于这些问题和不足，我们将在今后的工作中切实加以解决和改进。

2013年工作安排

今年是全面贯彻落实十八大精神的开局之年，也是我市推进转型发展的攻坚之年，机遇与挑战并存，工作十分关键。

面对今年的工作，我们在总体把握上作了两个基本判断：第一，现阶段的清远，转型发展与加快发展同样紧迫。清远经过十多年的快速发展，已经进入调整期，过去行之有效的粗放增长方式已经难以为继，加快转变经济发展方式、推动转型升级相当紧迫；但同时，经济发展总体不足、总量过小，人均水平过低，仍然是清远现阶段经济社会发展的主要矛盾。因此，必须按照省委胡春华书记在清远调研讲话的要求，把加快发展和转型升级有机结合起来，努力在加快发展中推动转型升级，通过转型升级更好地加快发展。第二，今年的发展环境依然偏紧，但也有不少有利因素。一方面，全球经济低速增长的态势仍将延续，国内经济增长的动力仍然不足，企业的经营环境依然困难，全市经济发展和资源环境硬约束的矛盾将进一步加剧。另一方面，我国新一轮的增长周期有望在全面建成小康社会的号角中提前来临；跨国公司和大型国企、民企正在酝酿新的产业布局调整；第三次工业革命浪潮初现，城镇化、工业化、信息化和农业现代化相互融合、共同推进的趋势将加快；珠三角特别是广州对环珠三角的辐射带动效应将更加明显，清远作为近水楼台，将获得更多的发展先机。特别要看到的是，省委、省政府作出了加快粤东西北发展的重大决策，将在交通等重大基础设施建设和城市扩容提质等方面，加大支持和投入的力度，这将为清远加快发展带来新的机会。

基于上述两个判断，今年的政府工作应该把握好五个关键点：第一，坚持科学地加快发展，通过提升增量来优化结构，既保持一定的增长速度，又防止片面追求速度而回到粗放增长的老路。第二，继续实施积极的财政策略，拓宽投融资渠道，用好用活社会资金，下最大的力气加快城市、产业发展平台和交通等基础设施建设。第三，全面系统深化改革，增创新的制度“红利”，突出解决经济领域市场化不足和社会领域市场化过度的问题，重点深化行政领域、社会领域和农村的综合改革。第四，加快推进与大广州地区发展的一体化，重点做好交通、产业和城市公共服务的对接与合作，努力打造环珠三角地区最优营商环境，提升接受珠三角辐射带动的能力。第五，高度重视民生和社会建设，千方百计增加城乡居民尤其是农村居民收入，优化财政支出结构，将更多的财力投向民生领域和社会事业。

基于上述研判和考虑，今年经济社会发展的主要预期目标是：国内生产总值增长8%；固定资产投资增长20%；地方公共财政预算收入增长7%；社会消费品零售总额增长12%；城镇居民可支配收入增长10%以上，农村居民人均纯收入增长12%以上，城镇居民登记失业率控制在3.5%以内，居民消费价格总水平上涨幅度控制在3%以内；城镇化率提高到50%左右；社会保障、资源环境保护、节能减排节水及安全生产完成省定目标。

与往年相比，尽管上述经济增长目标有所调低，但对于还处在调整转型期的清远来说，要实现上述预期增长目标，任务依然十分艰巨，需要我们加倍努力。

这里要特别说明的是，党的十八大提出了2020年全面建成小康社会的总体目标，省委也作出了2018年全省率先建成小康社会的决策。按照十八大的目标要求和国家统计局制定的《全面建设小康社会统计监测方案》进行测算，2012年清远全面小康社会的实现程度为78%，离全面小康目标还有22个百分点的差距。不仅远低于全省平均水平，也低于全国平均水平。从六大监测领域看，实现程度最低的是经济发展和文化教育，分别为62%和76.1%。要在2018年与全省一起率先全面建成小康社会，从2013至2018年，我市GDP的年均增长必须达到15.5%。清远目前还处于工业化的中期阶段，从国际和国内的经验看，这一阶段往往可以保持较高的经济增长速度，直至完成工业化。综合分析清远的区位优势、产业基础、资源条件和未来的发展机会，我们认为，只要全市上下一心，谋划得当，工作扎实，清远有条件也有可能，在未来六年实现GDP年均增长15.5%左右。但考虑到今年仍然处于调整期，经济步入新一轮快速增长区间的隐忧还没有完全消除，过去几年产业投入严重不足的实际，也难于支持更高的增长。因此，我们依然将今年GDP的增长目标确定为8%。我们希望通过今、明两年关键的工作，尽快缩短调整期，力争在今后几年实现有质量、有效益的快速增长，并始终把增加城乡居民收入、推进基本公共服务均等化摆到更加重要的位置。只有这样，我们才有可能如期建成全市人民真正认可的小康社会。

围绕实现上述年度目标，今年重点安排八项工作：

一、下大力气稳增长调结构

加快重点项目建设。设立市重点项目建设办公室，统筹协调推进全市重点项目的建设。把加快重点项目建设作为保增长调结构的重中之重，计划安排重点项目200项，总投资1900亿元，年度计划投资372亿元。完善重点项目审批“绿色通道”制度，优化项目前期审批的关键环节，促进项目尽快落地。建立市县两级班子成员联系督导、部门联席会议和项目建设管理考评机制，逐个项目明确前期工作完成时限，倒排项目建设工期，每两个月对重点项目进展情况进行全面检查，及时协调解决项目推进中遇到的问题。在加快续建项目的同时，确保新安排的项目前期工作上半年完成，下半年进入实质性施工阶段。

加大招商引资力度。明确全市招商引资方向和重点，制定招商引资年度行动计划。按照产业链垂直整合的招商思路，重点引进汽车及关键零部件、大食品、节能照明、生物医药和稀土材料等制造产业，大力引进金融、保险、电讯、旅游等服务产业的后台外包业务。今年要集中精力引进上述产业中的关键企业，再以关键企业为核心，引进上下游的关联企业，形成若干产业集群。以金融、保险为切入点引进呼叫产业，力争年内有一批关键企业入园建设，并由此入手发展电子商务。与广州联手发展空港经济，力争引进一批大型物流企业。着力引进有旅游开发经验的大企业，开发一批高端旅游项目。提高招商引资针对性，采取登门拜访、一对一沟通和小范围定向推介的方式，重点抓好广佛汽车及关联产业、深圳新上市企业、珠三角地区民营企业、有意愿拓展珠三角市场的内地大企业和日本、韩国及台湾地区相关产业的引进工作。协调好产业招商部门与县（市、区）、园区的工作关系，加强引进项目的衔接和统筹。

大力推进“扶优汰劣”。启动百家优质企业帮扶计划，逐家制订帮扶方案，逐个帮助解决项目立项、用地、用工等需要政府协助的问题，努力培育一批产值超十亿元、五十亿元和百亿元企业。认真落实各项优惠政策，深入推进结构性减税工作，进一步清理和减免行政事业性收费，着力解决好企业普遍碰到的问题，努力把清远打造成环珠三角最优的营商地区。制定政策逐步淘汰落后的陶瓷生产线和高新区产能落后企业，鼓励企业腾出空间发展物流产业和其他更有效益的产业；继续推进陶瓷、再生金属等传统产业转型升级，引导企业通过提高工艺、技术和装备水平，实现向制造环节两端延伸。

二、抓好事关长远的基础设施和产业平台建设

全力推进交通设施建设。把广清城际轨道作为融入珠三角的一号工程加快推进，确保上半年完成征地拆迁工作，为项目2016年完工创造条件。把握好今年全省启动高速公路建设大会战的历史性机遇，推动佛清从高速清远段今年上半年动工，连佛高速完成工程立项审批并着手征地拆迁，确保广清高速扩建清远段年内完成，广乐高速和二广高速清远段明年十月前完成。经过努力，省政府已同意把汕湛高速和汕昆高速清远段，提前安排到“十二五”建设，要抓紧与省相关部门沟通，加快前期工作，争取尽快动工。做好广清高速连接清连高速段的业主招标工作，尽快启动工程建设。加快国省道大修和改扩建，确保清三、清佛公路、106国道佛冈段大修工程和S354线太平至三坑段扩建工程主体完工，力争连怡、英坑等大修工程开工。尽快完成清连二级公路变更为国道的报批工作。加快推进北江千吨级航道、江南汽车客运站建设和清远港升级改造等工程。着手论证市区至广乐高速王子山出口连接线的可行性。

加快完善重点产业园区基础配套。完成莲湖产业园3200亩土地平整和“七通”建设，启动生活配套起步区建设，完善公交、教育、医疗等设施配套。加快雄兴污水处理厂建设。完成莲湖产业园二期和光电产业园征地工作。力争下半年启动并完成百嘉科技创新园、光电产业园土地平整和道路工程BT招标工作。加快“华南声谷”和源潭物流园的规划建设。进一步完善华侨工业园、广东顺德清远（英德）经济合作区、民族工业园等园区的配套建设。加快推进清新抽水蓄能、华侨工业园分布式能源项目、500千伏贤令山站主变扩容、220千伏清新石潭、库湾站配套线路等工程建设，强化园区发展的电力支撑。

加快打造“数字城市”。推进无线城市和“智慧城市”建设，尽快形成中心城市信息化设施与珠三角一体化的优势。加强与电讯运营商的合作，采取政府支持、企业参与的方式，逐步实现市区公共场所和部分营业场所免费无线上网。完成市级网上办事大厅建设并发挥效应，让市民群众通过电脑、手机办理和参与各类公共事务。加快数字城管网络平台建设，推进治安、交通、环境卫生管理等分平台建设，提升城市管理水平。建设全市教育、医疗的公共信息平台，推动市区、工业园区等重点区域宽带网络和3G移动信号全覆盖，为广大市民和投资者提供更加优质便利的信息服务。

三、加快“一心两核”的扩容提质

全面启动燕湖生态新城建设。按照建设生态新城的理念和指标要求，加快编制新城专项规划和分区控制性详细规划，启动大燕湖湖区工程、青榄海整治工程和一批市政道路、桥梁建设。上半年开工建设北江五桥及引道工程、黄腾峡大道、凤翔北路、凤翔南路南延段、北江东路、站前路、人民路东延段等一批骨干道路，年内完成学贤路东段、学府路等道路建设。加快北江四桥和燕湖新城一批新规划路网的前期工作，下半年开工建设清晖路等一批新区市政道路。同时，完善中心城区建成区路网，提升道路景观和配套水平。完成文化广场、静福广场改建工程，横荷、东城、乐排河等污水处理厂以及“两校一中心”等一批重点工程建设。完成市区第二、第三水厂前期工作，启动厂房建设，完成新的取水泵房工程和连接第一水厂的管道工程。按新的模式做好征地拆迁和农民安置工作，确保征拆一片，造福一片。

加快英德和连州新区建设。按中等城市规模规划建设英德、连州两个次中心城市。充分依托高铁站、广乐高速和顺德（英德）产业园、英红产业园，加快英德市区的扩容提质，加大英德市区与周边三镇规划建设的整合力度，推进城西片区和东岸新区的开发，形成一河两岸的大市区格局。依托二广高速加快连州市区的西扩北拓，融合深厚的历史文化底蕴彰显城市特色；推进连州新区建设与民族工业园开发的一体化，确保年内启动重点片区路网工程。在年度新增建设用地指标中安排专项，支持“一心两核”城市扩容提质。与此同时，加快其他县城和中心镇建设，不断完善基础配套和公共服务功能，增强集聚人口能力。

推进城市管理市场化。将清扫保洁、绿化养护、市政维修等职能推向市场。实施市容环境整治、数字化城管、绿化亮化提升、建筑工地环境整治、公路治超治限和城市水环境保护等“六大提升整治工程”，突出治理城市“六乱”现象，加大城中村整治力度，完善“门前三包”、24小时保洁等管理制度，加强日常巡查监管。积极探索推行垃圾分类处理、数字化、网格化等城市管理新模式，整合平安城市、智能交通、数字城管三大平台，构建城市综合监管系统，提高城市管理实效。

四、推进农村改革和农业现代化

以“三品”工程为抓手发展现代农业。巩固传统优势农产品，重点抓好沙糖桔黄龙病综合防控和流通体系建设，推进桂花鱼标准化生产，进一步扩大连州菜心和火龙果种植规模。加强名特优新品种的引进、培育和推广，力争每县引进和示范新品种3个以上，全市引进农业新品种15个以上，逐步将茶叶种植加工、草食动物和娃娃鱼养殖打造成为新的农业支柱产业。积极引导农业企业申报“三品一标”产品和农业名牌产品,年内新增绿色食品8个、有机产品5个、地理标志农产品2个、广东省名牌产品（农业类）2个、广东省著名商标2个，巩固发展食用农产品质量安全示范点76个。发挥农业产业化组织的作用，加大农产品品牌推广力度。大力扶持一批运营较为成功的农业专业合作社，发挥示范带动作用，推动新产业80%以上按照产业化模式组织生产。加快北部地区农网改造，推进145宗中小型灌区续建配套与节水改造、49宗机电排灌工程改造、3宗中小河流及10宗小流域治理等工程建设，改善农业生产条件。

从环境整治入手建设美丽乡村。深入实施农村清洁工程，大力推行禽畜集中圈养，垃圾“户收集、村集中、镇转运、县处理”。建立村民投入为主、村集体和政府补助为辅的筹资机制，落实人员、制度、经费，完善农村清洁卫生长效管理机制。大力整治村容村貌，全面铺开村道硬底化建设，全面清理、拆除农村闲置、废弃和私搭乱建住宅。在有条件的村庄实施整村完善改造工程，加大乡村绿化美化力度。深入开展文化、科技、卫生“三下乡”活动，大力普及环保、法制知识，引导村民移风易俗。鼓励开展“五好文明家庭”、“孝心示范户”、“好婆婆”、“好媳妇”等评选活动，积极开展关爱空巢老人和农村留守儿童活动，提高农村文明程度。通过美丽乡村建设，带动乡村旅游和农家乐的发展，增加农民收入来源。

丰富和深化农村综合改革。完善村民自治机制，充分发挥农村“长老”、“乡贤”的作用，在自然村建立村民理事会等社会组织，鼓励和支持社会组织参与村庄事务管理，试行以村民自治的方式管理村庄规划建设，逐步实现村庄事务的完全自治。在阳山、佛冈开展农村土地承包经营权的确权试点，建立农村土地流转交易平台，开展土地流转信息发布、政策咨询、合同鉴证、纠纷调解等服务工作，对土地承包经营权流转及集体建设用地使用权出让、出租，实行招标、拍卖、挂牌或网上竞价公开交易，为农业实现规模经营创造条件。创新农村金融服务，开展县级征信平台建设试点工作，建立县域统一的农户信用信息数据库。依托农民专业合作社，开展农户信用评分和信用等级评定，完善农户信贷服务。扩大农村抵押担保物范围，探索开展农村宅基地使用权和土地承包经营权抵押工作。

按照新标准推进扶贫开发。按照3300元的扶贫新标准，做好18万新贫困户的建档立卡工作，继续采取产业扶贫、智力扶贫、搬迁扶贫、金融扶贫等行之有效的帮扶措施，推进扶贫开发对口帮扶、干部驻村、财力支持、社会扶贫、贫困村班子建设五项工作长期化，帮助相对贫困村和贫困户脱贫。推进全国扶贫改革试验区建设，探索扶贫开发新经验。完成“两不具备”贫困村庄搬迁安置三年工作任务和低收入住房困难户住房改造建设年度任务。引导整村搬迁向城镇集中，推进“两不具备”贫困村居民市民化。妥善解决石灰岩地区外迁人口历史遗留问题。

五、提升基本公共服务均等化水平

缩小城乡区域教育发展差距。继续推进教育创强工作，确保年内全部县（市、区）建成省教育强县（市、区）、90%以上的建制镇（街）建成省教育强镇（街）。恢复偏远地区的教学点，完成25%的教学点改造。配齐配足农村中小学教师和教学仪器、设备设施，每个教学点至少配备2名教师。从今年起，用4年时间完成农村寄宿制学校改造，确保生活设施“五有”。采取补充专任教师、教师走教和远程同步教学等方式，开齐开足村小和教学点课程。着力提升北部地区办学水平，从今年起，市级教师培训经费和培训项目连续4年向北部农村教师倾斜；对各县（市）（不含县城所在镇）义务教育学校在编在岗正式工作人员按照人均每月不低于500元的标准发放岗位津贴。招募和选聘100名珠三角优秀退休教师到我市支教，选拔南部地区优秀教师到北部地区支教，安排北部地区教师到南部地区跟班学习，重点解决北部地区特别是少数民族地区优秀教师缺乏问题。推进各类教育统筹发展，建成乡镇中心幼儿园18所、村级幼儿园14所。按每生每学期200元的标准，补助普惠性规范化民办幼儿园。整体搬迁市第一中学，组建清远市高级中学，推动普通高中优质多样特色发展，实施全市中职跨区域招生、错位发展。推进省职教基地建设，力促岭南学院、国华学院等首批院校年内动工。推进市特殊教育学校二期和连州、阳山特殊教育学校建设，继续办好连阳4县（市）特教班。引导扶持民办学校上等级。

提升公共医疗卫生服务水平。加强基层医疗卫生机构建设，完成7间乡镇卫生院的业务用房标准化改建、257所村卫生站的标准化建设，用两年时间配齐基层医疗卫生机构基本医疗设备，启动镇村卫生服务一体化管理工作。加强县级医院建设，清城区人民医院和清新区人民医院达到二级甲等综合医院标准，英德市、连州市建成县级急救机构。按标准配备英德市中心血库。完善英德市和连州市并启动其它县（区）基层医疗卫生机构管理信息系统建设。建立等级医院对口协作基层卫生机构机制。加强人才建设，从今年起，每年从基层卫生院和社区卫生服务中心选送40名在职医师，进行全科医生培训；用3年时间，完成2072名注册乡村医生和卫生员的培训；采取“定点培训、协议就业”的方式，连续5年，每年招收100名大专以上应届毕业生到乡镇卫生院工作；招募和选聘珠三角优秀退休医生到我市支医。建立基层医卫人员待遇提升机制，今年实现与县级医疗机构相应人员待遇持平，明年起高于县级医疗机构相应人员待遇。

加快公共文化体育事业发展。启动城市文化工程，从根本上改变市中心城区文化建设严重滞后的状况。年内完成市中心图书馆、北江文化博物馆、城市展览馆、篮球综艺馆等项目的规划设计和前期工作，明年初开工建设，确保在本届政府任期内建成投入使用。改造市青少年宫和工人文化宫，完善配套功能。力争今年内完成市群众艺术馆、市民体育公园、江心岛市民文化艺术交流中心的建设。面向全国引进重大文化设施的管理运营人才，保障新建大型文化场馆的运营管理水平。启动乡村文化工程，以行政村为单位，逐步铺开乡村文化广场和乡村公园建设，配套建设篮球场和其他运动设施。大力推广乡村广场文化，由县、镇文化馆（站）组织人员到农村推广健身操和广场舞，丰富农村居民文化生活。完善农家书屋建设，增加农村实用技术类书籍。丰富提升校园文化、企业文化和机关文化，增进群众性文化交流。办好清远市第五届运动会。围绕“体现特色、提升形象”，整合各县（市、区）节庆文化资源，扎实筹办第八届广东国际旅游文化节。

六、在改善民生中加强社会建设

提升就业和社会保障水平。实施更加积极的就业和创业政策，力争城镇新增就业5万人，城镇失业人员再就业1.5万人。组织农村劳动力职业技能培训2.5万人，城乡居民创业培训4000人。抓好高校毕业生“三支一扶”计划的实施，组织好“南粤春暖”就业服务活动，抓好就业困难人员、零就业家庭就业工作。把农村劳动力技能培训与产业转型升级结合起来，大力开展企业员工在岗培训。落实积分制和高技能人才入户城镇政策，促进异地务工人员融入城市。完善均等化的社会保障公共服务体系，确保城乡居民养老保险参保率达到98%以上。进一步完善被征地农民养老保险制度。提高企业职工基本养老待遇水平，推行大病医疗救助和异地医疗费用实时结算。提升社会保险信息化水平，提高医保报销效率。探索建立医疗救助制度。加快发展老龄服务事业和产业，推进城市社区居家养老服务中心、服务站全覆盖，探索互助型农村养老服务模式。加强县级社会福利中心、乡镇敬老院建设。抓好保障性住房建设，确保完成省定任务。继续做好困难家庭住房、临时困难和灾害救助工作。适时启动低收入群众临时价格补贴机制，保障困难群众基本生活。继续做好市、县、镇三级肉菜市场的提升改造工作。

加强和创新社会管理。从行业协会类、公益慈善类、社会服务类社会组织着手，加快推进社会组织“五自四无”和“一业多会”改革。加大社会组织扶持力度，建立政府职能转移和购买服务制度，落实各项保障措施，拓宽社会组织参政议政渠道。建立健全利益分配机制，着力解决社会不公和机会不均等问题。以食品药品监管体系、企业融资征信体系和农民专业合作组织信用建设为切入点，以点带面铺开“两建”工作。探索完善食品安全监管体系，健全食品安全信息可追溯体系，大力治理“餐桌污染”。加大流动人口服务管理和特殊人群教育帮扶力度，推进社区矫正、青少年事务社工等工作。完善社会稳定风险评估和领导干部下访、接访、约访等制度，切实排查化解社会矛盾纠纷。完善社会治安防控体系，严厉打击违法犯罪活动。落实安全生产责任制，加大生产、交通、公共场所等领域的安全隐患排查治理，防止重特大安全事故的发生。做好“六五”普法和法律援助、法律服务、司法鉴定等工作。

投入12.3亿元办好十件民生实事。一是加大低保、五保救济力度，实现全市低保补差水平达到城镇每人每月250元，农村每人每月110元。二是推动农村公共服务下移，实现100%的现有社区居委会和90%的现有村委会建有公共服务站。三是完成“两不具备”贫困村庄搬迁安置4840户、22771人的任务。四是解决城乡困难家庭住房，安置1192户符合住房保障条件的城镇家庭，改造5745户农村低收入家庭住房。五是改善贫困地区医疗卫生条件，完成全市129条贫困村卫生站标准化建设，分批培训基层卫生骨干。六是大力开展劳动就业技能培训、人才交流和企业招聘会，解决4万人以上农村劳动力转移就业问题。七是发行和推广使用社会保障卡100万张，方便城乡居民办理社会保障及其他社会公共事务。八是按每生每年600元标准，对全市符合住宿条件的农村小学住宿生和初中农村住宿困难学生进行伙食补助；今后逐年提高补助标准。九是对全市44.42万亩基本农田进行高标准改造和建设。十是改善中心市区公共交通，调整和新增14条公交线路，新投放公交车不少于100辆。

七、扎实抓好生态环境的保护和建设

认真落实主体功能区规划。坚持因地制宜，实施差异化发展。南部地区在发展高端产业、现代服务业的同时，加大节能减排和生态修复力度；北部地区在加强生态建设和环境保护的同时，探索生态优势转化为经济优势的有效途径，发展适合本地实际的绿色产业。进一步探索和完善资源要素配置、财政转移支付、生态补偿等政策机制，使土地指标和相关发展要素更多向重点开发区倾斜，财政转移支付更多向生态发展区倾斜。结合大部制改革，减少生态发展区财政供养人口数量，降低公共支出压力。

抓好环境保护和污染综合治理。把生态环境作为最核心的竞争力，切实加大“市肺”工程、林业重点生态工程建设力度，加强自然保护区建设管理，对笔架山、飞霞山等中心城区郊野公园进行规划控制。积极开展生态村、生态镇等生态示范创建活动，逐步推进“绿色社区”、“绿色学校”、“绿色教育基地”等基层绿色创建活动。大力整治非法开采、乱采乱挖林地行为，抓好地质灾害防治工作，加大水土保持力度，加快矿山和生态脆弱区植被恢复。鼓励企业通过技术升级实现结构减排，加大重点行业环境整治，促进高污染、高排放行业转型升级。严格环评审批，拒绝高污染、高耗能项目落户。彻底排查饮用水源地及主要江河流域污染源，深入开展水环境整治专项行动，重点抓好北江、连江流域水资源保护，严格控制江河上游工业园区项目准入。扎实推进农业污染源减排，防治禽畜养殖业污染。加强城市机动车排气污染防治，实施机动车区域限行，加快淘汰“黄标车”。推行“绿色施工”，控制和减少工地扬尘污染。大力推进陶瓷行业脱硫、水泥行业脱硝等工作。严格落实固体废物管理制度，建立固体废物处理全过程监管体系。

集约节约利用资源。实行最严格的耕地保护制度，突出加强基本农田保护，确保耕地数量不减少、质量不降低。积极推进集约节约用地，抓好开发强度、建筑密度、容积率、绿地率等指标控制，保障重点建设项目和民生工程用地需求。继续整顿和规范矿产资源开发秩序，限制和淘汰粗放型资源开采项目。大力发展循环经济，推进矿产资源、固体废弃物资源综合利用以及再生资源、水资源的循环利用。深入推进企业节能行动，开展建筑节能示范，积极创建节能型机关，促进交通、商业、民用等领域节能推广。推行企业节水生产和农业节水灌溉，确保用水总量、用水效率、水功能区限制纳污等指标控制在省定目标范围内。

八、以高效廉洁为目标抓好政府建设

围绕提升效能深化政府改革。整合市级机构和人员编制，使部门审批事项向单个科（股）室集中，人员力量从审批岗位向监管岗位倾斜。坚持效率优先，在规范运作的同时，采取更多提高效率的方法。全面完成市、县两级大部门制改革，实现政府效能的提升和职能的转变。启动并加快推进事业单位“去行政化”改革。进一步清理和规范行政审批事项，公布第二批市级政府行政审批事项改革目录。继续推进非税和基金管理改革，实施“零基预算”管理，提高财政资金使用绩效。

围绕重点工作落实强化作风建设。建立领导班子和公务员定期学习制度，倡导深入扎实的调查研究。强化为民意识，把清远人民对幸福生活的追求，作为政府工作的最大目标。大力推行现场办公，多开有效的协调会，认真落实首问责任、限时办结制度。强化政府工作的分解落实，明确目标进度，定期开展督查督办。严格落实中央“八项规定”，进一步精简会议和文件，大力推行办公自动化和视频会议，改进文风会风。针对“正风”行动和“公述民评”反映的突出问题，加大明查暗访力度，建立责任追究机制，集中排查解决基层和社会反映强烈的机关作风问题。完善公务员竞争上岗及激励机制，激发各级干部，尤其是中层干部的责任感和事业心。加强与上级部门的沟通对接，争取更多资源支持清远发展。

围绕优化政务环境推进廉政建设。完善网络问政平台及政务信息共享平台，拓宽政务公开渠道，通过政府发言人制度的常态化运作，实现政府与社会的信息对称，让政府更好地接受社会公众监督。更自觉地接受人大、政协的监督，并作出制度化的安排。健全廉政风险防控体系，将行政执法、财政资金运行、工程和土地交易、社保基金、住房公积金等纳入电子监察。支持审计机关依法独立开展工作，加强重点建设、民生领域和领导干部经济责任审计。切实树立过“紧日子”的思想，推进部门预算公开，从紧安排财政支出，严控“三公”经费开支，将更多资金用于改善民生。

此外，要深入开展创建“双拥”模范城活动，积极支持国防和部队建设，加强国防动员、民兵预备役和人防、反走私工作。认真落实妇女儿童发展纲要，继续抓好民族宗教、残疾人、统计、外事、侨务、对台、气象、地震、档案、方志等工作。

各位代表，清远的发展已经进入关键时期，今年的工作具有决定性意义。让我们在省委和市委的坚强领导下，以更扎实高效的作风，用更多的智慧和汗水，同心协力，迎难而上，认真落实好今年的各项工作，为清远今后几年更好更快的发展奠定坚实的基础，为全面建成小康社会，实现全市人民共同的清远梦而努力奋斗！
